# *PIF* Genes Mediate the Effect of Sucrose on Seedling Growth Dynamics

**DOI:** 10.1371/journal.pone.0019894

**Published:** 2011-05-23

**Authors:** Jodi L. Stewart, Julin N. Maloof, Jennifer L. Nemhauser

**Affiliations:** 1 Department of Biology, University of Washington, Seattle, Washington, United States of America; 2 Department of Plant Biology, University of California Davis, Davis, California, United States of America; Ecole Normale Superieure, France

## Abstract

As photoautotrophs, plants can use both the form and amount of fixed carbon as a measure of the light environment. In this study, we used a variety of approaches to elucidate the role of exogenous sucrose in modifying seedling growth dynamics. In addition to its known effects on germination, high-resolution temporal analysis revealed that sucrose could extend the number of days plants exhibited rapid hypocotyl elongation, leading to dramatic increases in ultimate seedling height. In addition, sucrose changed the timing of daily growth maxima, demonstrating that diel growth dynamics are more plastic than previously suspected. Sucrose-dependent growth promotion required function of multiple phytochrome-interacting factors (PIFs), and overexpression of *PIF5* led to growth dynamics similar to plants exposed to sucrose. Consistent with this result, sucrose was found to increase levels of PIF5 protein. PIFs have well-established roles as integrators of response to light levels, time of day and phytohormone signaling. Our findings strongly suggest that carbon availability can modify the known photomorphogenetic signaling network.

## Introduction

As a plant emerges from the seed, it must make an accurate and nuanced assessment of the light environment. Light-directed development, or photomorphogenesis, is marked by establishment of photosynthetically-competent embryonic leaves (cotyledons) optimally positioned towards a light source by the embryonic stem (hypocotyl) [1]. Hypocotyl elongation contributes to the positioning of cotyledons largely through differential cell elongation–in *Arabidopsis*, hypocotyl epidermal cells can elongate up to 100 times their embryonic size [2]. Levels of photosynthate reflect the seedling environment, and transportation of fixed carbon from source to sink cells is essential for this growth. Over the course of every day, the form and abundance of carbon is adjusted to meet the plant's metabolic needs [3]. During the day, fixed carbon is primarily stored as starch in the chloroplasts of photosynthetically-active cells. At night, starch is converted into sucrose which travels from the leaves into the rest of the plant. Expression of starch degrading enzymes is circadian regulated [4], [5], allowing plants to anticipate future carbon demands. The degradation of starch is highly correlated with growth and is tightly regulated to prevent the plant from exhausting its resources [6]. Indeed, plants have been shown to rapidly adjust their starch accumulation strategy to take best advantage of changing light conditions [7].

In addition to stimulating production of photosynthate, light inhibits hypocotyl elongation through activation of photoreceptors, primarily the red-light absorbing phytochromes (phys) and blue-light absorbing cryptochromes (crys) [8]. Over the past 30 years, genetic screens have implicated more than two dozen factors downstream of photoreceptor function [9]. A number of recent studies have focused attention on one group of these proteins, a family of light labile basic helix-loop-helix (bHLH) transcription factors called phytochrome interacting factors (PIFs). Several of the PIFs have been shown to directly interact with light-activated phytochromes and subsequently be targeted for degradation [10]. The PIFs have varying dimerization and phytochrome-binding characteristics and have been shown to regulate separate aspects of photomorphogenesis [11]. For example, *PIF1*, *PIF3*, *PIF4*, and *PIF5* contribute to hypocotyl elongation, while *PIF1* and *PIF6* regulate seed germination [12], [13], [14]. Plants lacking *PIF1*, *PIF3*, *PIF4*, and *PIF5* function, called *pifq* mutants, phenocopy morphological and transcriptional responses of light-grown plants even when grown in complete darkness [15], [16]. PIF proteins act in part through regulating phytohormone pathways, including auxin and gibberellins [17], [18], [19], [20], [21], [22], [23].

How the very large number of factors influencing seedling growth are integrated is a complex problem that remains to be solved. Time-lapse imaging studies suggest that growth can be partitioned into discrete regulatory modules. For example, blue light inhibition of hypocotyl elongation can be separated into short-term growth slowing and longer-term maintenance phases, each under the control of different blue light receptors [24], [25], [26]. Genetically distinct phases of growth cessation and maintenance have also been reported for ethylene responses [27]. To understand the molecular mechanisms of these regulatory modules, periods of sensitivity must be defined for each factor that regulates photomorphogenesis.

In this study, we found that sucrose could alter many seedling growth parameters, including: germination, growth duration, and maximal growth rate. In addition, the presence of sucrose could dramatically shift daily growth rhythms of hypocotyl elongation. Sucrose promotion of growth required the function of several members of the PIF family of transcription factors. Surprisingly, growth dynamics of plants exposed to sucrose could be partially mimicked by overexpression of *PIF5*. While sucrose did not dramatically alter expression of any of the *PIF* genes, sucrose treatment did result in higher levels of PIF5 protein. Together, our results place the sensing of carbon availability in the same PIF-mediated growth network as photoreceptors, the circadian clock and phytohormones.

## Results and Discussion

### Sucrose promotes seedling growth by extending the number of days of hypocotyl elongation

**Figure 1:**
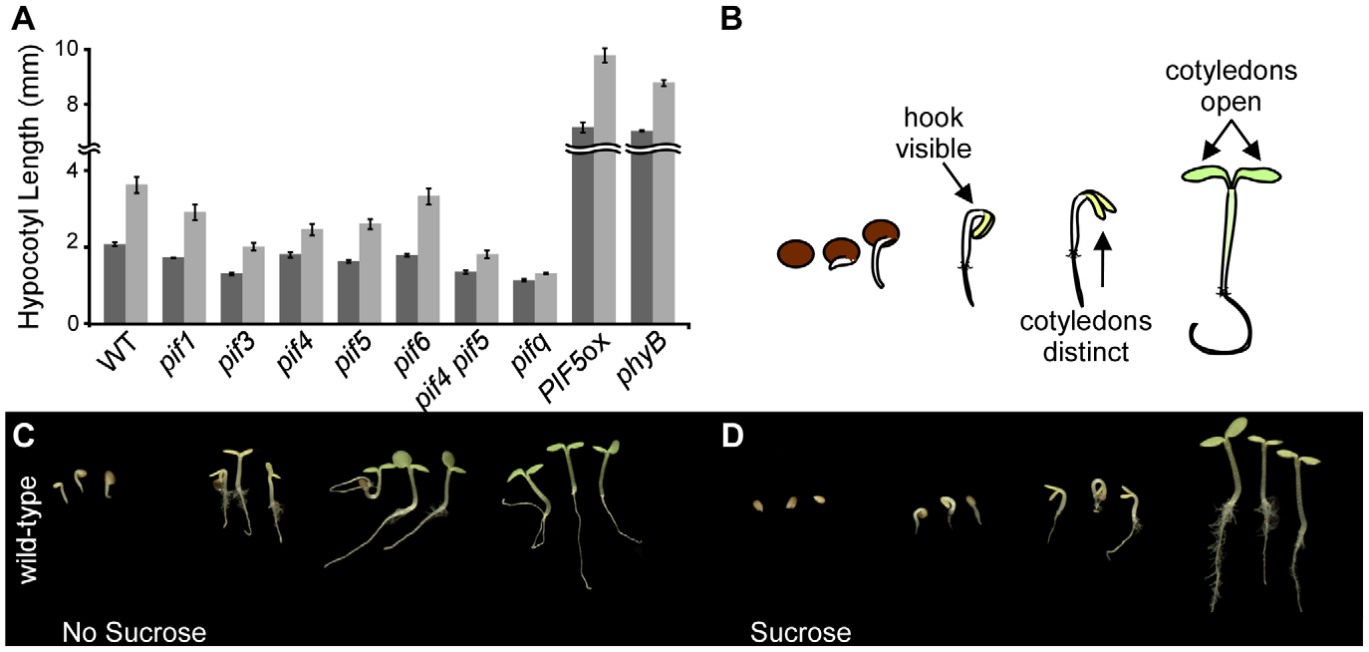
Sucrose promotion of hypocotyl elongation requires activity of *PIF* genes. (A) By six days, wild-type (WT) seedlings grown on 88 mM (3%) sucrose (light bars) were taller than seedlings grown without sucrose (dark bars). *pif3*, *pif4*, and *pif5* seedlings showed significantly reduced response to sucrose with further reductions observed in *pif4 pif5* mutants. Sucrose response was almost completely eliminated in *pifq* mutants lacking *pif1 pif3 pif4* and *pif5* function. Overexpression of *PIF5* (*PIF5*ox) resulted in elongated hypocotyls in the absence of exogenous sucrose and significantly enhanced growth promotion with added sucrose. *phyB* mutants where PIF5 levels are known to be increased resemble *PIF5*ox seedlings without sucrose, but show a wild-type response to sucrose. Error bars represent standard error for at least two independent experiments with 15–20 seedlings of each genotype in each experiment. Asterisks indicate significantly different responses between tested genotype and wild type (* = p<0.05, ** = p<0.01, *** = p<0.001) using a linear regression. Note the broken y-axis. (B) After emergence, the radicle extended, tightly-hooked cotyledons became visible, and collet hairs at the hypocotyl/root junction appeared. Cotyledons then became distinct from one another, opening until the maximum angle between the cotyledons was reached. (C–D) Three representative seedlings are shown at the dawn of day 3, 4, 5 and 6 in each panel. (C) Wild-type seedlings entered the labeled stages shown in (B) by the dawn of day 3, 4 and 5, respectively. (D) Addition of sucrose to the growth media resulted in the hook becoming visible approximately one day later (day 4). Sucrose also caused a modest delay in cotyledon opening. Seedlings were grown in short-day conditions in 30 µmol m^−2^ sec^−1^ white light.

The addition of 88 mM (3%) sucrose to plant media nearly doubled the height of six day old seedlings (Figure 1A), while causing a delay in germination (Figure 1C,D), consistent with previous reports [28], [29], [30], [31]. Addition of comparable levels of mannitol caused a strong reduction in overall hypocotyl elongation (Figure S1A), and had no effect on timing of germination (Figure S1B,C), suggesting that sucrose effects were not the result of changes in osmotic potential. Given these observations, we hypothesized that sucrose must alter growth rate and/or duration of growth to cause dramatically increased final hypocotyl lengths despite a shorter growth period.

**Figure 2:**
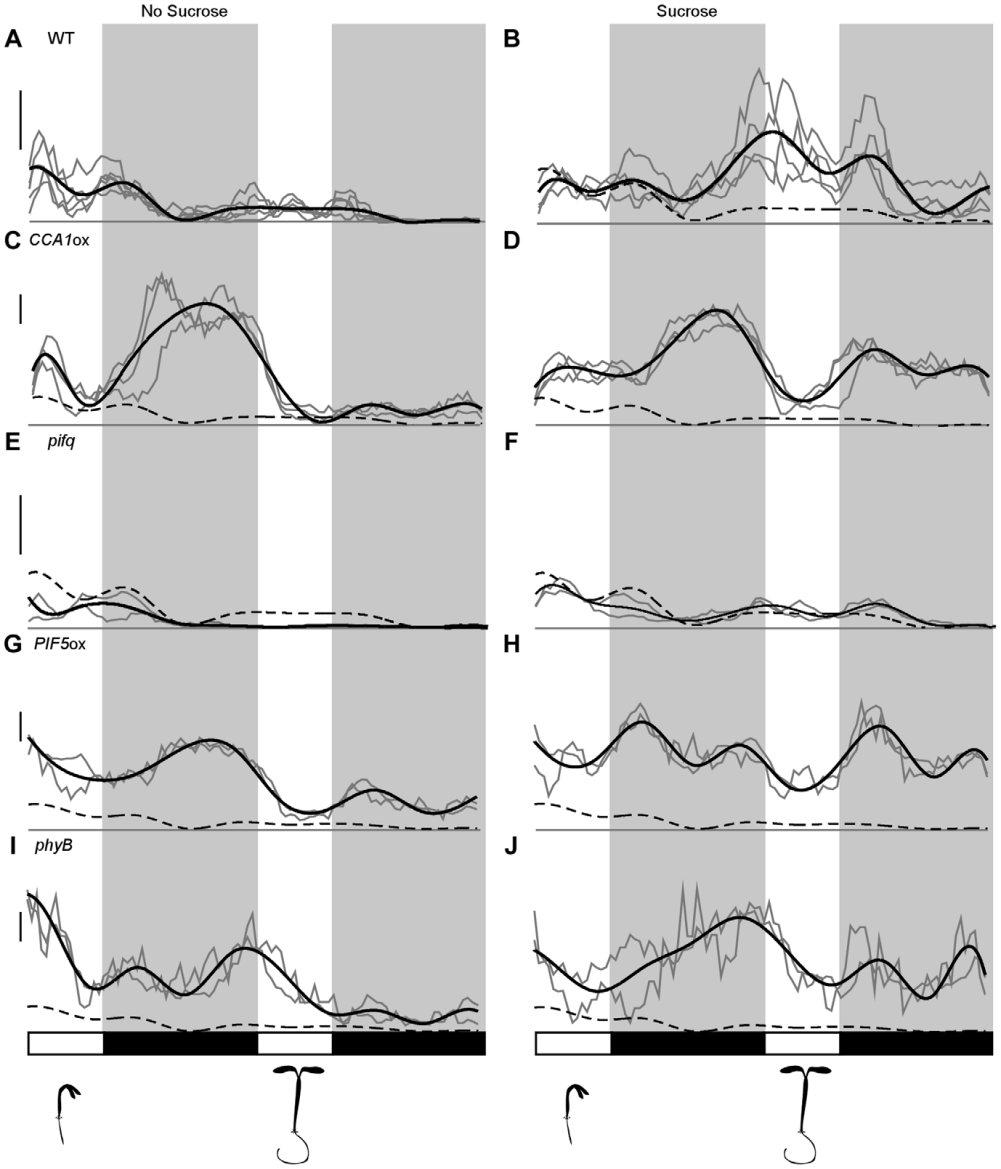
Sucrose requires *PIF* function to extend the number of days of seedling growth. (A) Wild-type hypocotyl elongation rates diminished after the cotyledons opened. (B) Addition of sucrose caused sustained high growth rates. (C, D) In *CCA1*ox, sucrose caused a similar increase in duration of high hypocotyl elongations rates. (E) *pifq* hypocotyls had lower average growth rates but similar dynamics to wild type. (F) In *pifq* mutants, sucrose had substantially reduced effects on later stage hypocotyl elongation rates. (G, H) Hypocotyls of *PIF5*ox seedlings had elevated early and late elongation rates without exogenous sucrose (G) but showed enhanced sensitivity to exogenous sucrose (H). (I, J) *phyB* mutants showed similar growth rates to *PIF5*ox mutants with (I) and without (J) sucrose. Each independent experiment is shown in grey and growth rates represent an average of 15–20 seedlings. Smoothed average growth rates are shown in black. A dashed black line representing wild-type growth rates without sucrose is shown for reference. Light and dark phases are indicated in the bars below the graphs. Schematic representations of seedling stages shown below the graphs are accurate for all seedlings, except *PIF5*ox (G,H). *PIF5*ox seedlings show an enhanced developmental delay phenotype, further exaggerated by addition of sucrose (Figure S3). Scale bar equals 0.05 mm/hr.

To test this hypothesis, we assessed seedling growth rate using time-lapse imaging. Our measurements were synchronized to begin when seedlings had a visible apical hook (shortly after stage 0.5 as described in [32]). Hypocotyls were then measured at 30 minute intervals for three subsequent days. For plants grown in standard media without sucrose, distinct hypocotyl elongation dynamics were observed for each of the developmental stages highlighted in Figure 1B. After the apical hook became visible, hypocotyls exhibited low but consistent levels of elongation during the day and first half of the night followed by a gradual rise in growth rate towards dawn (Figure S2). As the cotyledons became distinct from one another, growth rate spiked at dawn (Figure 2A). By the time the cotyledons were fully open, growth rates were at the lowest levels overall with small, waning growth peaks at day/night transitions (Figure 2A).

Sucrose addition caused the most dramatic change in growth dynamics in seedlings with fully opened cotyledons, a time when seedlings grown without sucrose had largely stopped growing. In contrast, plants grown with exogenous sucrose showed sustained strong rhythmic growth patterns (Fig. 2B), reminiscent of patterns observed in previous studies [33]. This growth extension phenotype was further exaggerated in plants over-expressing *CIRCADIAN CLOCK ASSOCIATED 1* (*CCA1*ox) (Figure 2C,D), where reduced circadian clock function causes rapid growth throughout the entire night period [33]. Extra growth phases were not a result of osmotic effects of sucrose, as mannitol did not alter seedling growth dynamics (Figure S1D). Thus, sucrose addition leads to taller seedlings by prolonging the duration of growth in combination with a modest increase in growth rate. This extended growth period allows sucrose treated seedlings to overcome their early developmental delay.

### Sucrose and other environmental factors can change timing of maximal daily growth

**Figure 3:**
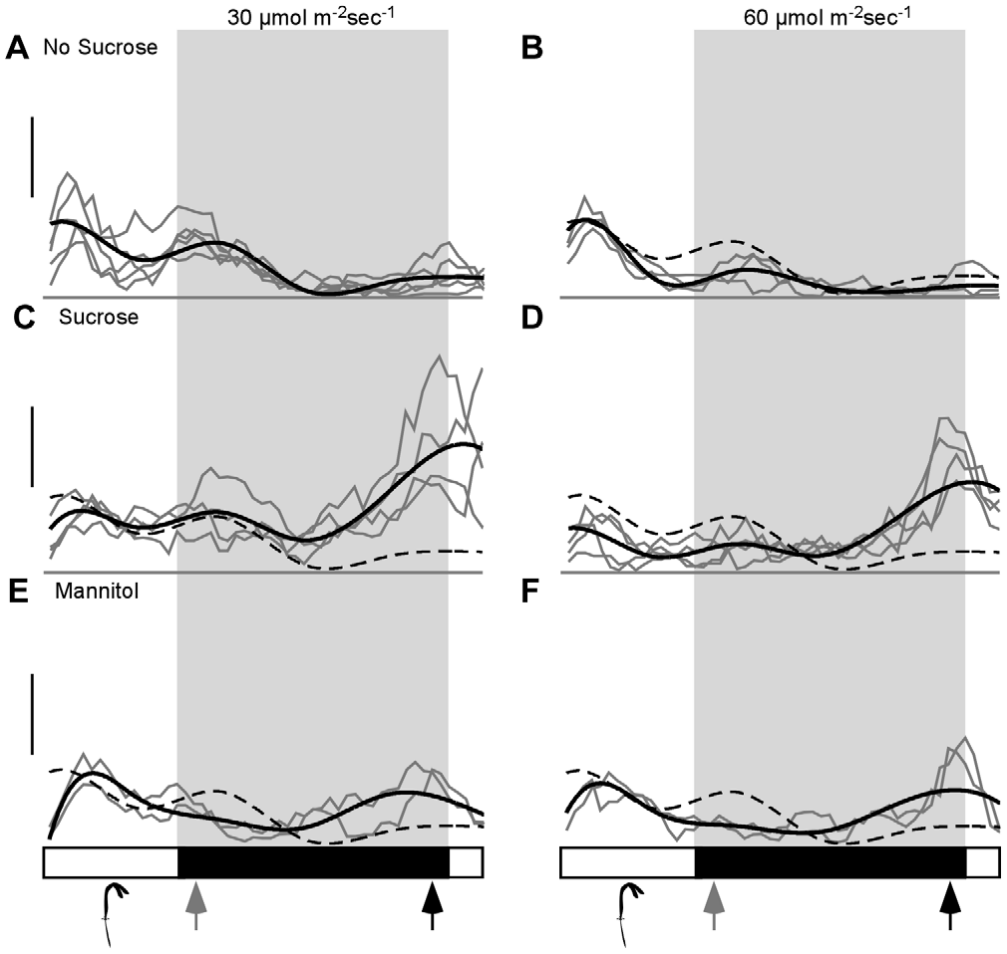
Diel patterns of rapid hypocotyl elongation phases are highly plastic. (A) Hypocotyl elongation occurred at dusk (grey arrow) and dawn (black arrow) in our standard light conditions (30 µmol m^−2^ sec^−1^). (B) Growth rates were lowered by increased light intensity (60 µmol m^−2^ sec^−1^), most notably at dusk. (C) Seedlings grown on sucrose showed higher rates of hypocotyl elongation. This was particularly evident at dawn. (D) When sucrose and higher light intensity (60 µmol m^−2^ sec^−1^) were combined, both the reduced growth rate at dusk and the increased growth rate at dawn were observed. (E) Addition of mannitol caused increased hypocotyl elongation at dawn, similar to the effects of sucrose albeit with lower magnitude. (F) Higher light intensity (60 µmol m^−2^ sec^−1^) reduced growth rates in mannitol, as was observed in other conditions. Each independent experiment is shown in grey and growth rates represent an average of 15–20 seedlings. Smoothed average growth rates are shown in black. A dashed black line representing wild-type growth rates without additives is shown for reference. Light and dark phases are indicated in the bars below the graphs. Schematic representations of seedling stage are shown below the graphs. Scale bar equals 0.05 mm/hr.

The external coincidence model for rhythmic growth proposed by Nozue et al. relies on two independent effects on the key growth regulators PIF4 and PIF5: the circadian system regulates gene expression and light alters protein stability [33]. In our experiments, plants grown in all conditions showed an initial burst of rapid dawn elongation once cotyledons were distinct (Figure 2). On media without sucrose (Figure 3A), subsequent periods of rapid elongation occurred at both dawn and dusk with decreasing amplitude. This is in contrast to the sustained and predominant dawn growth peaks previously reported for seedlings grown on sucrose [33].

Addition of sucrose increased growth rates, particularly at dawn (Figure 3C, black arrow, dashed versus solid line). This pattern began to resemble the previously published growth pattern by Nozue and colleagues, although a small growth peak at dusk could still be detected in our conditions (Figure 3C, grey arrow). When light intensity was increased from 30 µmol m^−2^ sec^−1^ to 60 µmol m^−2^ sec^−1^ (the conditions used in Nozue et al., 2007), total hypocotyl elongation was reduced. The greatest decrease in growth rate was observed at dusk (Figure 3B, grey arrow, dashed vs. solid lines). These results demonstrate that both light levels and sucrose addition can act independently to change the distribution of growth throughout the day. When both conditions are used, their combined effects are essentially additive (Figure 3D, dashed vs. solid lines). This highlights the critical importance of assessing growth rates in each new condition, as additional factors may also shape ultimate growth patterns. Interestingly, addition of mannitol could partially reproduce the effects of sucrose on daily growth peaks, although with substantially lower maximum growth rates (Figure 3E,F). This suggests that unlike early developmental delays, sucrose effects on diel growth rhythms were at least partially through altered osmotic potential. Previous studies have shown that *PIF4* and *PIF5* transcripts can be high at both dusk and dawn [33], [34], [35], creating symmetrical potential growth windows. These two windows for PIF activity are further supported by the strong dusk growth peaks observed in continuous light conditions [36]. Our results suggest that the timing of rhythmic hypocotyl elongation is plastic and can be altered with subtle changes in growth conditions, including light intensity and media formulation. While our plants were all grown in artificial laboratory conditions, it is likely that variations in natural environments resulting in altered photosynthetic or developmental rates could lead to similar changes in growth patterns.

### Growth promoting effects of sucrose require PIF function

It is well-established that sucrose can interfere with light responses [37], [38], [39]. PIF transcription factors contribute to hypocotyl elongation [16], [40], chlorophyll biogenesis [41], and seed germination [12], [13], [14], [41]—all of which are also affected by exogenous sucrose. To test whether sucrose was acting through the *PIF* family, we grew a number of single and multiple *PIF* mutants with and without sucrose.

In conditions without added sucrose, hypocotyl phenotypes of *pif* mutants matched previous reports (Figure 1A) [11]. When sucrose was added to the media, the growth promotion responses of *pif3*, *pif4* and *pif5* were significantly diminished compared to wild type (Figure 1A). As *PIF4* and *PIF5* have been shown to act partially redundantly in rhythmic hypocotyl growth [33], we also examined *pif4 pif5* double mutants. Loss of both *pif4* and *pif5* caused a further reduction in sucrose response, and additional loss of *PIF1* and *PIF3* function in the *pifq* mutant nearly eliminated sucrose promotion of growth (Figure 1A). Hypocotyls of *pifq* mutants grew more slowly than those of wild-type plants and for fewer days (Figure 2E). While sucrose could still cause modest growth of older *pifq* seedlings, average growth rates remained substantially lower than wild-type plants (Figure 2F).

If sucrose was acting through PIF proteins, we reasoned that higher levels of PIF activity might phenocopy sucrose effects. To test this, we focused on *PIF5*, as loss of *PIF5* function is known to have the most dramatic effect on rhythmic hypocotyl elongation of single loss-of-function *pif* mutants [33]. *PIF5*ox seedlings were taller than wild-type seedling grown on sucrose and showed a statistically enhanced response (Figure 1A). Moreover, even in the absence of exogenous sucrose, *PIF5*ox seedlings showed substantial late growth (Figure 2G). This late growth rate was higher than that observed in *CCA1*ox seedlings (Figure 2C,D vs. 2G,H), suggesting that the phenotype of *PIF5*ox plants was not solely the result of defects in clock function.

### Sucrose increases levels of PIF5 protein

**Figure 4:**
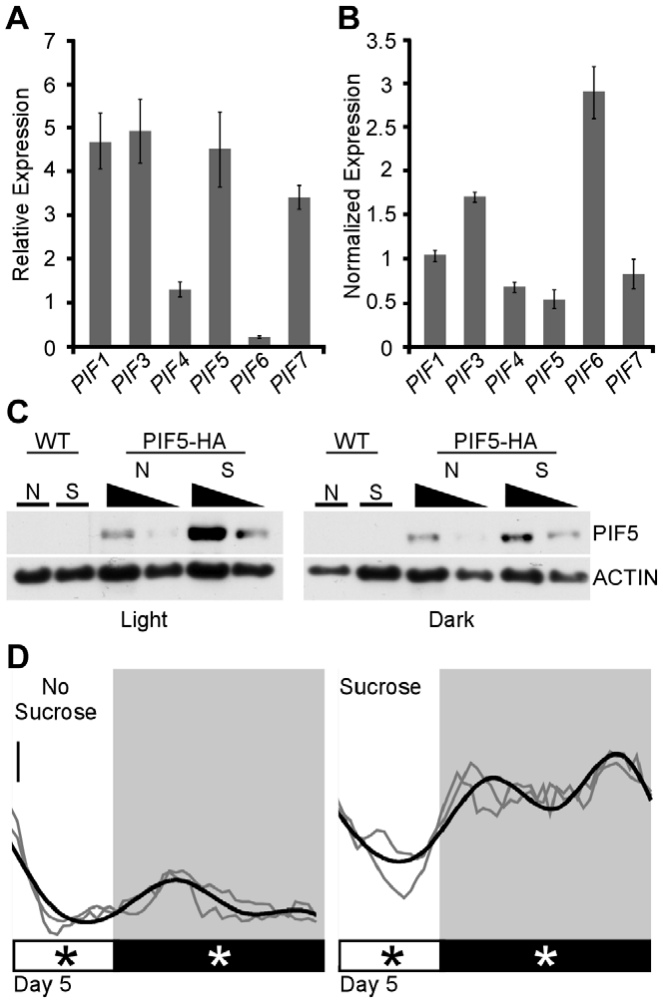
Sucrose effects on seedling growth dynamics are likely mediated by stabilized PIF proteins. (A) Expression of *PIF* genes at midnight on day 5 is shown for the no sucrose condition in *CCA*ox seedlings. Expression values are shown relative to a control gene. Error bars represent the standard error from three biological replicates. (B) Relative values are shown for *PIF* gene expression in plants grown on sucrose normalized to expression in the no sucrose condition (shown in A). Sucrose had only modest effects on *PIF* gene expression. *PIF3* and *PIF6* were induced by sucrose, while *PIF4* and *PIF5* were slightly repressed. Seedlings were collected 8 hours after lights off (midnight) on day 5 (no sucrose) or day 6 (with sucrose) to match developmental stage. Error bars represent the standard error from three biological replicates. (C) Sucrose increased PIF5-HA levels in both light and darkness. Wild-type or 35S::PIF5-HA seedlings [46] were collected 4 hours after lights on (Light) or 8 hours after lights off (Dark) on day 5. Anti-HA antibodies were used to detect PIF5-HA proteins (upper panel) and anti-ACTIN antibodies were used as a loading control (lower panel). Two concentrations (approximately 1X and 0.5X) are shown for each PIF5-HA sample. “N” and “S” indicate the no sucrose and sucrose treatments, respectively. Note that overall levels of protein are higher in light samples, as indicted by increased signal in the loading control. The blots shown here are representative of at least two experiments with independent biological replicates. (D) High growth rates continue into day 5 for 35S::PIF5-HA seedlings supplied with exogenous sucrose. Each independent experiment is shown in grey and growth rates represent an average of 15–20 seedlings. Smoothed average growth rates are shown in black. Light and dark phases are indicated in the bars below the graphs. Asterisks indicate collection times for protein abundance assays. Note that *PIF5*ox seedlings have developmental defects, making it impossible to align collections by developmental stage (Figure S3). Scale bar equals 0.05 mm/hr.

*PIF* family members are under transcriptional and post-translational control [42]. To test sucrose effect on *PIF* expression, we extracted mRNA from *CCA1*ox seedlings grown with or without exogenous sucrose (Figure 4A,B). The *CCA1*ox background was used to attenuate potentially confounding effects of the circadian clock on *PIF* gene expression. Sucrose had no effect on expression of *PIF1* or *PIF7*, caused a modest increase in expression of *PIF3* and *PIF6*, and led to a slight decrease in expression of *PIF4* and *PIF5* (Figure 4B, Table S1). The small effects on *PIF3*, *PIF4*, and *PIF5*—the genes with the largest loss-of-function effects on sucrose promotion of growth—suggest that sucrose is unlikely to alter growth dynamics through transcriptional control of *PIF* genes.

To test for sucrose effects on PIF protein, we grew HA-tagged *PIF5*ox seedlings [33] with or without sucrose (Figure 4C). These lines had similar growth dynamics and sucrose sensitivity as the untagged *PIF5*ox lines (Figure 4D), including a strong developmental delay (Figure S3) making it impossible to match developmental stages. We found that when comparing seedlings of the same age, sucrose dramatically increased PIF5 abundance in both light and dark periods (Figure 4C), although the effect was strongest in the light. One possible mechanism for this increase in PIF5 levels is through reduced function of phyB, which is known to destabilize PIF5 protein [43]. Surprisingly, *phyB* null mutants showed a wild-type response to sucrose (Figure 1A, Figure 2I,J), suggesting that sucrose effect on PIF activity is phyB-independent. It is possible that other factors, such as closely related phytochrome family members, may take over phyB's role in its absence.

The morphological transformations of photomorphogenesis are happening concurrently with major shifts in metabolism. Given that sucrose is synthesized and transported throughout the plant, it is possible that exogenous sucrose may conflict with the seedling's own photosynthesis-derived signals. Our results suggest a model where light-directed degradation of PIF protein is antagonized by high carbon availability. Previous work [44], in combination with the results presented here, suggest that adding sucrose to the media may have a similarly dramatic effect on photomorphogenesis as phytohormone treatments. Sucrose dependency on PIF function provides direct molecular integration of photoreceptor and phytohormone signal transduction pathways with a yet-to-be-determined carbon-sensing mechanism.

## Materials and Methods

### Plant materials and growth conditions

Wild type is *Arabidopsis thaliana* ecotype Col-0. *CCA1*ox (also known as *CCA1-34*) [45], *pif4* (*pif4-101)* [46], *pif5* (*pil6-1*) [47], *pif4 pif5* [46], *PIF5*ox (PIF5-OXL2) [47], HA-tagged *PIF5*ox [46], and *phyB-9* (also known as *hy3-EMS142*) [48] are as previously described. *pif1* (also known as *pil5-1*) [12] and *pif6-2* [13] were provided by G. Choi (Korea Advanced Institute of Science and Technology) and K. Halliday (Edinburgh University), respectively. *pif3-3* [49] and *pifq* [40] were provided by P. Quail (University of California, Berkeley). Seeds were sterilized for 20 min in 70% ethanol, 0.01% Triton X-100, followed by a rinse in 95% ethanol. After sterilization, seeds were suspended in 0.1% agar (BP1423, Fisher Scientific) and spotted on plates containing 0.5X Linsmaier and Skoog (LS) (LSP03, Caisson Laboratories, Inc.) with 0.8% agar. Sucrose (S2, Fisher Scientific) and D-mannitol (69-65-8, Acros Organics) treatments were performed by mixing 88 mM of either additive into the media before sterilization. Seeds were then stratified in the dark at 4°C for 3 days. Plates were placed vertically in a Percival E-30B growth chamber set at 20°C in 30 or 60 µmol m^−2^ sec^−1^ white light. All plants were grown in short-day conditions (8 hours light, 16 hours dark) and placed in the growth chamber at dawn.

### Microscopy and time-lapse photography

Time-lapse photography is essentially as described in Nozue et al. (2007), Images were captured every 30 minutes by a charge-coupled device camera (PL-B781F, PixeLINK) equipped with a lens (NMV-25M1, Navitar) and IR longpass filter (LP830-35.5, Midwest Optical Systems, Inc.). Image capture was accompanied by a 0.5 second flash of infrared light by a custom built LED infrared illuminator (512-QED234, Mouser Electronics). A custom LabVIEW (National Instruments) program controlled image capture and illumination. Color seedling images were collected at 10X magnification using a Leica dissecting scope (S8APO, Leica Microsystems) and camera (DFC290, Leica Microsystems).

### Hypocotyl measurements

For end-point analysis, hypocotyl lengths were measured from 12–25, 6-day old seedlings per treatment by scanning vertical plates using ImageJ software (http://rsb.info.nih.gov/ij/). For growth rate analysis, hypocotyl lengths from at least 12 individuals were measured using ImageJ software for each time-lapse image (2208×3000 pixels). Growth rates were calculated from hypocotyl lengths using a custom script in MATLAB (MathWorks), available on request.

### RNA extraction and qRT-PCR analysis

Seedlings were grown vertically in three rows on 0.5X LS plates with 2% agar. *PIF* expression analysis was performed on *CCA1*ox seedlings collected 8 hours from lights off (midnight) on day 5 for plates without sucrose and on day 6 for plates with sucrose to match developmental stage. Roots were manually removed at the time of collection. Samples were collected using a light equipped with a green filter (LS139, Acey Decy Equipment Co., Inc.). All samples were immediately frozen in liquid nitrogen and stored at −80°C until processing. Total RNA was extracted from tissue of approximately 1000 seedlings using the Spectrum Plant Total RNA Kit (Sigma), total RNA was treated with DNaseI on columns (Qiagen) and 1 µg of eluted RNA was used for complementary DNA (cDNA) synthesis using iScript (Biorad). Samples were analyzed using SYBR Green Supermix (Biorad) reactions run in a Chromo4 Real-Time PCR system (MJ Research). Expression for each gene was calculated using the formula (E_target_)^−ΔCPtarget(control−sample)^/(E_ref_) ^−ΔCPref(control−sample)^ [50] and normalized to a reference gene (At1g13320).

### Western blot analysis

PIF5-HA abundance was detected in extracts of whole *PIF5HA*ox and wild-type seedlings collected 4 hours from lights on (midday) or 8 hours from lights off (midnight) on day 5. Total protein was extracted from approximately 100 mg of tissue using the method described in [51], except that anti-HA-peroxidase (Roche) was used at a 1∶1000 dilution. Samples were loaded at two concentrations (1X and 0.5X) to better estimate relative abundance. Anti-ACTIN antibodies (A0480, Sigma) were used at a 1∶2000 dilution and detected with anti-Mouse (172-1011, Biorad) used at a 1∶20,000 dilution. SuperSignal West Femto Maximum Sensitivity Substrate (Pierce) was used to detect signals.

## Supporting Information

Figure S1 **Mannitol does not increase growth or delay early seedling development.** (A) By day 6, seedlings grown on mannitol were significantly shorter than those grown on standard media. Error bars show standard error for three experiments with 12–25 six day old seedlings in each experiment. Asterisk indicates significance (Student's t-test: p<0.05). (B, C) Addition of mannitol did not alter seedling progression through development (B). Wild-type seedlings grown on standard media are the same as those shown in Fig. 1C. Three representative seedlings are shown at the dawn of day 3, 4, 5 and 6. (D) Duration of rapid hypocotyl elongation was not sensitive to mannitol. Each independent experiment is shown in grey and growth rates represent an average of 15–20 seedlings. Smoothed average growth rates are shown in black. A dashed black line representing wild-type growth rates without mannitol is shown for reference. Light and dark phases are indicated in the bars below the graphs. Schematic representations of growth stages are shown below the graph. Scale bar equals 0.05 mm/hr.
(TIF)

Figure S2 **In their earliest phase, hypocotyls showed low but consistent rates of elongation.** Each independent experiment is shown in grey and growth rates represent an average of 15–20 seedlings. Smoothed average growth rates are shown in black. Light and dark phases are indicated in the bars below the graphs. Dawn of day 3 is shown. Schematic representation of growth stage is shown below the graph. Scale bar equals 0.05 mm/hr.
(TIF)

Figure S3 ***PIF5*** **ox seedlings were developmentally delayed with and without sucrose.** (A, B) Wild-type seedlings are the same as those shown in Fig. 1C. (C) *PIF5*ox seedlings were delayed in cotyledon opening. (D) The *PIF5*ox developmental delay phenotype was exaggerated in the presence of sucrose. Three representative seedlings are shown at the dawn of day 3, 4, 5 and 6.
(TIF)

Table S1 **Analysis of** ***PIF*** **expression in response to sucrose.** Above ground tissue was collected at midnight of day 5 from *CCA1*ox seedlings without sucrose, and day 6 from those grown with sucrose. Expression values were calculated using the formula (E_target_)^−Cttarget^/(E_ref_) ^−Ctref^ where E is the primer efficiency and the reference gene is At1g13320. Primers are shown in the 5′ to 3′ direction. ^a^Relative expression of tissue collected from seedlings grown without sucrose. ^b^Relative expression of tissue collected from seedlings grown with sucrose. ^c^P-values calculated using Student's t-test to compare no sucrose and sucrose treatments.
(DOC)
