# Supplementary figures and images for: PIF Genes Mediate the Effect of Sucrose on Seedling Growth Dynamics
Source: PLoS One. 2011 May 23;6(5):e19894. doi: 10.1371/journal.pone.0019894 (PMC3100310; doi:10.1371/journal.pone.0019894)

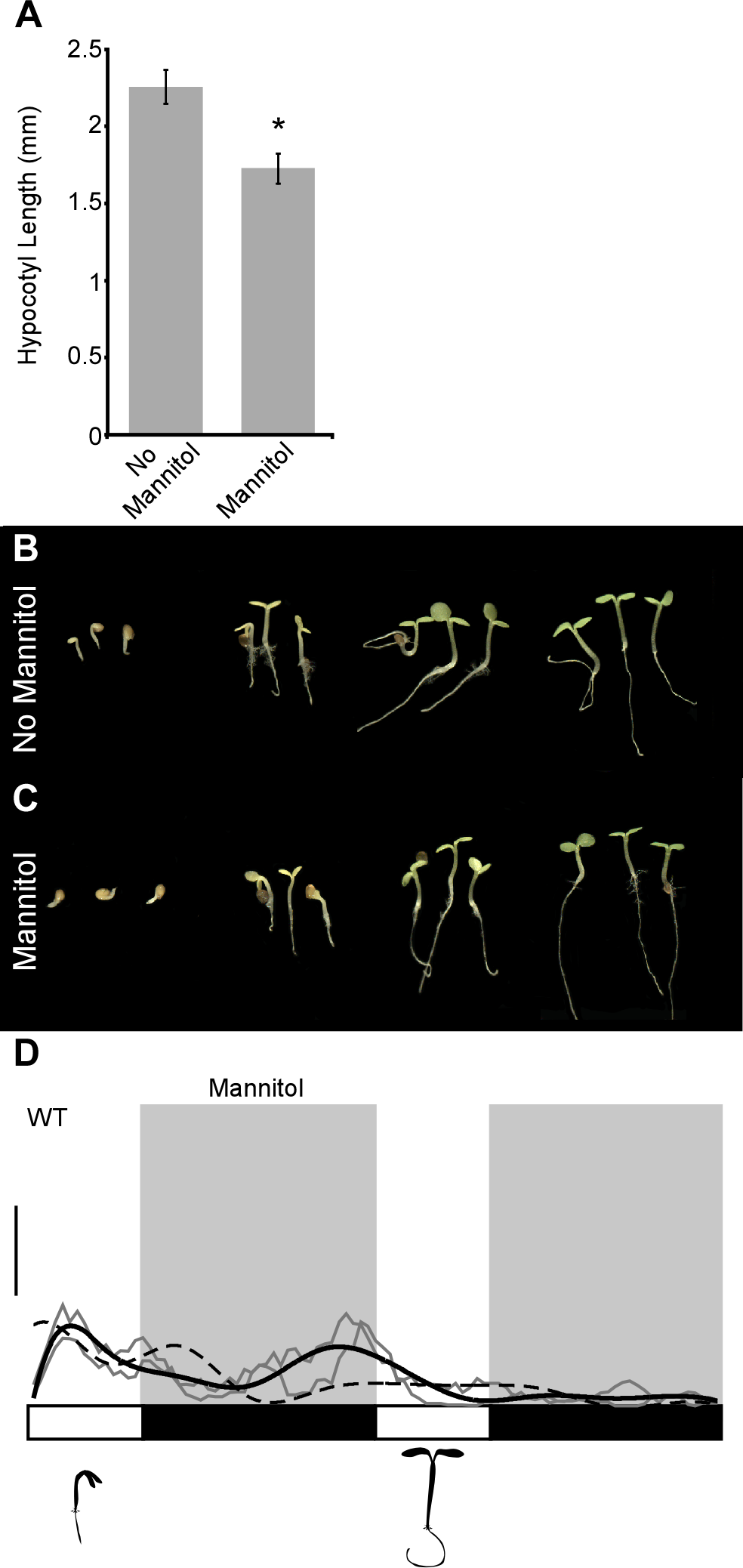

Supplement: Figure S1 — Mannitol does not increase growth or delay early seedling development. (A) By day 6, seedlings grown on mannitol were significantly shorter than those grown on standard media. Error bars show standard error for three experiments with 12–25 six day old seedlings in each experiment. Asterisk indicates significance (Student's t-test: p<0.05). (B, C) Addition of mannitol did not alter seedling progression through development (B). Wild-type seedlings grown on standard media are the same as those shown in Fig. 1C. Three representative seedlings are shown at the dawn of day 3, 4, 5 and 6. (D) Duration of rapid hypocotyl elongation was not sensitive to mannitol. Each independent experiment is shown in grey and growth rates represent an average of 15–20 seedlings. Smoothed average growth rates are shown in black. A dashed black line representing wild-type growth rates without mannitol is shown for reference. Light and dark phases are indicated in the bars below the graphs. Schematic representations of growth stages are shown below the graph. Scale bar equals 0.05 mm/hr. (TIF) [file pone.0019894.s001.tif]

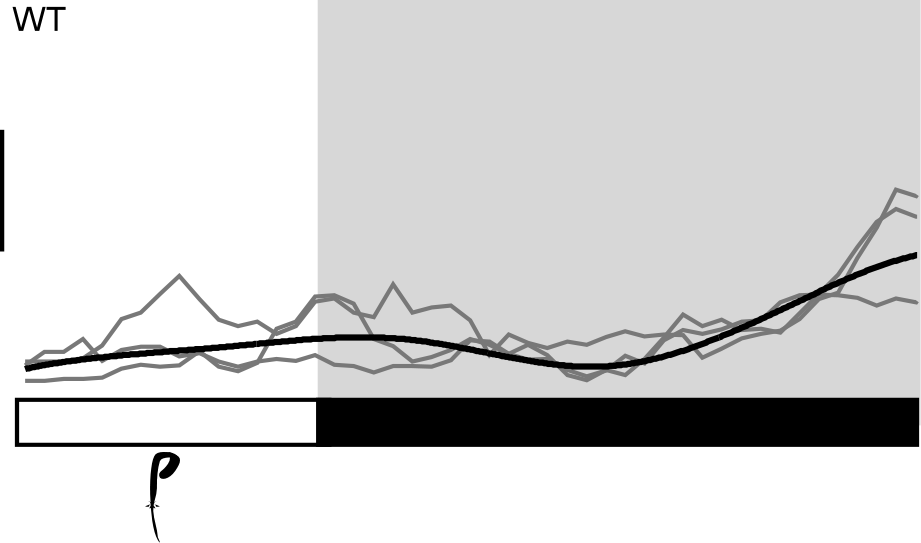

Supplement: Figure S2 — In their earliest phase, hypocotyls showed low but consistent rates of elongation. Each independent experiment is shown in grey and growth rates represent an average of 15–20 seedlings. Smoothed average growth rates are shown in black. Light and dark phases are indicated in the bars below the graphs. Dawn of day 3 is shown. Schematic representation of growth stage is shown below the graph. Scale bar equals 0.05 mm/hr. (TIF) [file pone.0019894.s002.tif]

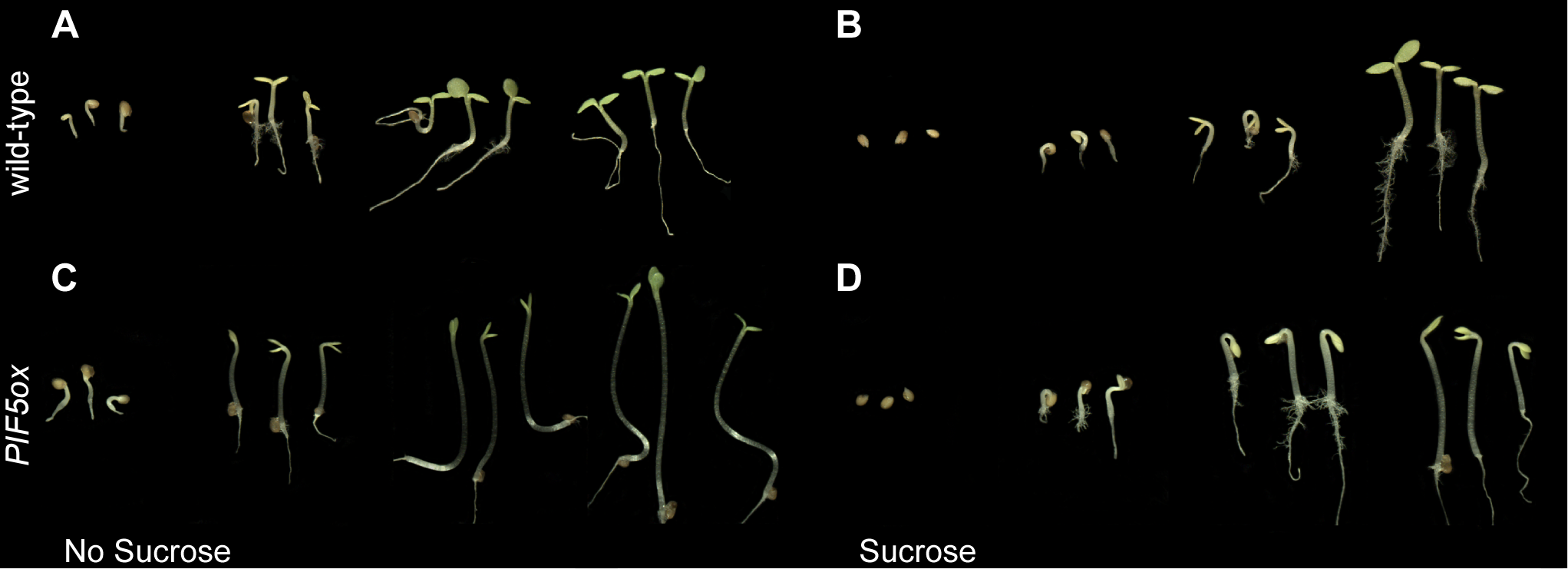

Supplement: Figure S3 — PIF5 ox seedlings were developmentally delayed with and without sucrose. (A, B) Wild-type seedlings are the same as those shown in Fig. 1C. (C) PIF5ox seedlings were delayed in cotyledon opening. (D) The PIF5ox developmental delay phenotype was exaggerated in the presence of sucrose. Three representative seedlings are shown at the dawn of day 3, 4, 5 and 6. (TIF) [file pone.0019894.s003.tif]
