# Supplementary material for: PIF Genes Mediate the Effect of Sucrose on Seedling Growth Dynamics
Source: PLoS One. 2011 May 23;6(5):e19894. doi: 10.1371/journal.pone.0019894 (PMC3100310; doi:10.1371/journal.pone.0019894)
Supplement: Table S1 — Analysis of PIF expression in response to sucrose. Above ground tissue was collected at midnight of day 5 from CCA1ox seedlings without sucrose, and day 6 from those grown with sucrose. Expression values were calculated using the formula (Etarget)−Cttarget/(Eref) −Ctref where E is the primer efficiency and the reference gene is At1g13320. Primers are shown in the 5′ to 3′ direction. aRelative expression of tissue collected from seedlings grown without sucrose. bRelative expression of tissue collected from seedlings grown with sucrose. cP-values calculated using Student's t-test to compare no sucrose and sucrose treatments. (DOC) [file pone.0019894.s004.doc]

| **Gene #** | **Gene Name** | **No Sucrose^a^** | **Sucrose^b^** | **P-value^c^** | **Forward Primer** | **Reverse Primer** |
| --- | --- | --- | --- | --- | --- | --- |
| At1g09530 | *PIF3* | 4.92 ± 0.72 | 8.29 ± 0.93 | 0.05 | CCTAGCCAACAAACCAATCC | AGTGGTTCCTGCGTCTGAGT |
| At2g20180 | *PIF1* | 4.68 ± 0.63 | 4.80 ± 0.61 | 0.89 | CCCGTCAAGAGTCTTTGTACC | CCCGAGGTTGGATCATACTG |
| At2g43010 | *PIF4* | 1.28 ± 0.17 | 0.86 ± 0.10 | 0.10 | GCCGATGGAGATGTTGAGAT | GACGGTTGTTGACTTTGCTG |
| At3g59060 | *PIF5* | 4.50 ± 0.85 | 2.25 ± 0.13 | 0.06 | AAAACCCGGTACAGTTGCAG | GCTGCTCCGATAAGATTTGG |
| At3g62090 | *PIF6* | 0.18 ± 0.03 | 0.51 ± 0.04 | <0.01 | TGGACTAATGCCAATGCAAA | TTTTCCGTTGGAATGAAAGG |
| At5g61270 | *PIF7* | 3.38 ± 0.27 | 2.74 ± 0.41 | 0.26 | GGTGGTGGAAATGGTTATGG | GACCATCATTGGTGGAGGAG |
